# Integrating Digital Health Technologies With Physical and Mental Health for Motivating Low-Income Older Adults

**DOI:** 10.1093/geroni/igaf122.1146

**Published:** 2025-12-31

**Authors:** Ladda Thiamwong, Chitra Banarjee, Kworweinski Lafontant, Jethro Raphael Suarez, Abigail Tice, Dahee Kim, Michael Dino, Rui Xie

**Affiliations:** University of Central Florida, Orlando, Florida, United States; University of Central Florida, Orlando, Florida, United States; University of Central Florida, Orlando, Florida, United States; University of Central Florida, Florida, United States; University of Central Florida, Orlando, Florida, United States; University of Central Florida, Orlando, Florida, United States; University of Central Florida, University of Central Florida, Florida, United States; University of Central Florida, Orlando, Florida, United States

## Abstract

Digital health technology can play a transformative role in improving overall well-being and the accuracy of frailty prediction. Digital health interventions enhance physical activity (PA) across various chronic conditions and improve depression. and mental health outcomes. However, limited research indicated that digital health technology may benefit populations experiencing health disparities by enhancing accessibility and engagement, which can reduce adverse outcomes. We examined the associations among digital health technologies, physical function, and depression in low-income older adults aged 60+ using data from our clinical trial study funded by NIH (R01MD018025). Digital health technologies, including sensor-based technologies (Balance Tracking System/BTrackS for static balance (SB), wearable accelerometers (ActiGraph) for physical activity (PA), and a mobile application with Ecological Momentary Assessment (EMA) to measure motivation for PA using a single slider-style question. Physical function was assessed using dynamic balance (Timed Up and Go: TUG) and lower limb strength (Sit-to-Stand: STS). Depression was assessed using Patient Health Questionnaire-9 (PHQ-9), and frailty was measured using the FRAIL questionnaire. We found both measures of physical function (TUG and STS) predicted motivation for PA (pTUG < 0.001, pSTS = 0.054). We also found that depression significantly predicted motivation for PA (pPHQ < 0.001), suggesting that motivation for PA is driven by both physical capabilities and mental health. On the other hand, frailty did not significantly correlate with PA motivation (p = 0.471). Integrating digital health technologies that offer reminders and progress tracking can motivate low-income older adults to stay active and enhance physical function and mental health.

